# Excessive Daytime Sleepiness and Epilepsy: A Systematic Review

**DOI:** 10.1155/2013/629469

**Published:** 2013-10-31

**Authors:** Andre S. Giorelli, Pâmela Passos, Thiago Carnaval, Marleide da Mota Gomes

**Affiliations:** ^1^Riosono Sleep Disorders Clinic, Rua Siqueira Campos 53 Sala 1104, Copacabana, 22031-070 Rio de Janeiro, RJ, Brazil; ^2^Epilepsy Program, Deolindo Couto Neurology Institute, Universidade Federal do Rio de Janeiro, Av. Venceslau Braz 95, Botafogo, 22290-140 Rio de Janeiro, RJ, Brazil

## Abstract

*Background*. Sleep complaints are common in patients with epilepsy (PWE). Excessive daytime sleepiness (EDS) is one of the most reported complaints and its impact is still a matter of debate. *Objective*. Evaluate the relationship between EDS and epilepsy, with emphasis on prevalence, assessment, and causes. *Methods*. A systematic review on PubMed database in the last 10 years (2002 to 2012). The search returned 53 articles and 34 were considered relevant. After citation analysis, 3 more articles were included. *Results*. Most studies were cross-sectional and questionnaire based. 14 papers addressed EDS as the primary endpoint. 14 adult and 3 children studies used subjective and objective analysis as methodology. The number of studies increased throughout the decade, with 21 in the last 5 years. Adult studies represent almost three times the number of children studies. EDS prevalence in PWE varies from 10 to 47.5%. Prevalence was higher in developing countries. *Conclusion*. EDS seems to be related more frequently to undiagnosed sleep disorders than to epilepsy-related factors, and although it affects the quality of life of PWE, it can be improved by treating comorbid primary sleep disorders.

## 1. Introduction

Sleep disorders are now recognized as a major impairment in quality of life and work productivity. These complaints are especially common in patients with epilepsy (PWE) normally with more severe consequences than in the general population [1]. Fragmented or inadequate sleep can exacerbate daytime drowsiness and memory dysfunction, which are already present in epilepsy either because of the pathological substrate or the use of antiepileptic drugs (AEDs) and also contribute to intractable seizures [1]. There is a cycle created by sleep disruption leading to worsening seizures, which in turn leads to an even greater impairment of sleep [1]. One of the most reported sleep-related complaints in PWE is excessive daytime sleepiness (EDS) and it has been mainly interpreted as a side effect of AEDs treatment and frequent seizures [2, 3]. This does not seem to be the case. Several studies have shown that EDS in PWE is comparable to that of controls and that symptoms suggestive of obstructive sleep apnea (OSA) and restless legs syndrome (RLS) are stronger predictors of subjective daytime sleepiness than frequency of seizures or AED [2, 3].

One of the problems faced by studies addressing EDS specifically is that a uniform operational definition is still lacking, probably because it is not a disease or a disorder but a symptom presented in primary sleep disorders, such as narcolepsy, OSA, and RLS. The International Classification of Sleep Disorders 2nd edition (ICSD-2) defines EDS as *“the inability to stay awake and alert during major waking episodes of the day, resulting into unintended lapses into drowsiness or sleep”* [4]. Since EDS has a subjective interpretation, it could be mistaken as tiredness or fatigue, thus presenting a diagnostic challenge [5].

EDS can be studied by subjective or objective evaluation. From a subjective point of view, structured questionnaires and scales that are validated in the general population are commonly used. The Epworth Sleepiness Scale (ESS) is the most common instrument used in sleep research [6]. It is a quick self-applied scale with 8 typical dozing situations with a 0 to 3 point score for each question. With a maximum of 24 points, scores >10 are considered abnormal for most authors [6, 7] and it has been validated to Brazilian Portuguese [8]. For the objective evaluation, the multiple sleep latencies test (MSLT) and maintenance of wakefulness test (MWT) are commonly used. Although these tests were developed to address daytime sleepiness in hypersomnia syndromes, they also have been widely used to study sleepiness in other situations. The tests should be performed on the following day of a full night polysomnography (PSG). The patient is given five 20-minute opportunities to sleep (MSLT) with a 2-hour interval between the recordings. The mean sleep latency is calculated and values <10 minutes are considered abnormal. For the MWT, four 40-minute opportunities to stay awake are given and a mean sleep latency <8 minutes is considered abnormal [9]. Other types of objective evaluation for EDS are pupillometry, cognitive, and psychomotor function, evoked potentials, and alpha attenuation test (AAT). Of those, pupillometry and long latency cortical and cognitive evoked potentials are less used either because of lack of studies demonstrating its clinical utility or by large degree of intersubject variability [10]. Cognitive and psychomotor tasks are used to test the effects of sleep disruption on cognitive and psychomotor performance. They can also be used to assess response to treatment, but they only evaluate the effects of sleepiness on the efficiency of brain function and do not measure sleepiness directly. AAT is based on changes of the alpha frequency that occur on the transition from wakefulness to sleep. Patients are instructed to open and close their eyes 8 times, with each movement lasting 1 minute, while being in an illuminated room. AAT correlated significantly with MSLT, even higher than subjective instruments [10]. 

The objective of this review is to analyze the current evidence on EDS and epilepsy, with emphasis on prevalence, assessment, and causes.

## 2. Methods

We performed a systematic review to identify all articles addressing EDS in PWE. A PubMed database search was performed for all publications existing and “in press,” in the last 10 years (October 2002 to October 2012). The search terms used were *“sleepiness” OR “sleep disruption” OR “disturbed sleep” AND “epilepsy” AND “sleep*.*”* The keywords were restricted to title and abstract. No language restriction was made and citations of relevant studies were checked. Exclusion criteria included review papers and articles that do not use a validated questionnaire for subjective analysis as part of the research methodology. The search returned 53 articles that were reviewed and 34 articles were selected. After citation analysis, three more articles were added because of their relevance [2, 3, 11].

## 3. Results

Of the 37 articles selected, 27 were adult-based and 10 were children-based studies. Regarding the study design, the articles were 23 cross-sectional studies, 11 prospective cohort studies, 2 case reports, and one case series.

### 3.1. Evidence from Adult-Based Studies

Most of the questionnaire-based studies addressed the prevalence of either EDS or primary sleep disorders.  Table 1 summarizes these studies. Evidence shows that sleep disorders (especially OSA and insomnia) are twice to 3 times more common in PWE than in controls [12–14]. On the other hand, the data on EDS is not so straightforward, with studies showing that the prevalence is similar to controls [2, 3, 12, 13] and others reporting a high prevalence in PWE [14–18]. An ESS score >10 was reported in 18 to 47% of PWE and 12 to 17% of controls [2, 15, 16, 18, 19], with a trend for higher ESS scores in patients with intractable seizures [17]. The most common sleep-related complaints in PWE were maintenance insomnia and EDS [13, 14, 16]. Sleepiness in PWE may have a multifactorial origin and studies show that symptoms of OSA and RLS are independent predictors of an ESS score >10 [2, 3, 20]. This has been also demonstrated in patients of older age and by the fact that the treatment of OSA with continuous positive airway pressure (CPAP) can improve EDS and seizure control [21]. Another study prospectively analyzed the effect of CPAP treatment in 29 epilepsy patients with OSA diagnosed on PSG and with EDS evaluated with the ESS. The median followup was 26 months and 52% had an ESS score >10. In 21 patients, OSA symptoms coincided with a clear increase in seizure frequency or the occurrence of status epilepticus. Patients with good CPAP compliance had a significant reduction in EES score and in seizure frequency [22]. In contrast, a study with 125 PWE determined the cut-off of the sleep apnea scale of the sleep disorders questionnaire (SA-SDQ) in this population and suggested that in PWE the values should be lower than in the general population and that EDS may not be a good predictor in epilepsy as in the general population [23]. Very few studies addressed EDS in PWE with subjective and objective analysis. A study conducted in Brazil evaluated EDS with ESS and PSG/MSLT in 39 patients with temporal lobe epilepsy (TLE). The most frequent complaints were daytime sleepiness (85%), frequent awakenings (79%), and nocturnal seizures (69%), 13% had OSA, and ESS scores correlated with MSLT mean latencies. EDS was found in 36% (ESS score >10) [24].

Another common finding is the relationship between EDS and depression or anxiety in PWE. A study with 247 patients showed that anxiety, depression, and sleep disturbance had greater effect than seizure control in quality of life (QOL) scores and also found underdiagnosis of psychiatric comorbidity in PWE [25]. EDS complaints were reported in 47.5% of 99 PWE and correlated with anxiety and neck circumference [19].

Although previous reports suggest that seizure type, AEDs, and nocturnal seizures could contribute to disrupted sleep and EDS, the presence of primary sleep disorders seems to play a significant role in this complex interaction. Studies have demonstrated that nocturnal seizures could disrupt sleep structure and even brief seizures could result in prolonged alterations in sleep architecture that lasts longer than the postictal period and their treatment results in improvement of sleep parameters [1]. This effect could help explain why patients with only nocturnal seizures have daytime complaints of sleepiness. On the other hand, some studies suggest that nocturnal seizures were not significant predictors of EDS [2, 13, 16, 26]. In one study of patients with nocturnal frontal lobe epilepsy (NFLE), EDS was similar between patients and controls, although daytime symptoms of sleepiness could be more frequent in a subgroup of patients with higher ESS scores, irrespective of seizure frequency [26]. In a study evaluating sleep disturbances in patients with refractory and controlled epilepsy with subjective and PSG evaluation, EDS and OSA were more frequent in the refractory group, and it also found shorter average sleep time in contrast to self-reported longer sleep time in this group of patients, suggesting that OSA and sleep fragmentation could have a negative impact in seizure control [17].

The role of AEDs in EDS has also been studied and the evidence shows that patients with a stable medication regimen do not report more EDS [18, 19]. A study with levetiracetam showed that it can initially reduce sleep time and disturb sleep architecture, but as the treatment proceeded this effect was less important [27], and there was a report of a patient with severe but potentially reversible hypersomnia without subjective EDS, with levetiracetam as an add-on treatment [28]. The effects of topiramate and pregabalin on daytime sleepiness have also been studied. 14 patients were prospectively studied with a short monotherapy course with topiramate 200 mg/day and it did not impair daytime vigilance assessed by MSLT and visual reaction time [29]. Pregabalin, in a prospective study with 12 patients, improved seizure control, increased REM sleep, and reduced stage N2. There was also an increase in ESS score but within the normal limits, suggesting mild daytime sleepiness [30]. Another prospective double-blind, randomized study evaluated the effect of pregabalin 300 mg/day versus placebo on polysomnographic variables in 17 patients with well controlled partial seizures and reported sleep disturbance. The pregabalin group showed improvement in sleep continuity with reduction of awakenings and improvement in wake time after sleep onset, and also improvement in subjective scales [31].

The role of epilepsy surgery has also been addressed by some studies. On a retrospective review of 21 patients treated with resective surgery for drug-resistant focal epilepsy (NFLE), 9 had complaints of EDS was preoperatively and after surgical treatment all patients were seizure-free and EDS resolved in those who previously reported it, despite the maintenance of AED treatment, suggesting that the arousal instability that caused sleep fragmentation and EDS was related to recurrent epileptic discharges not detectable on scalp EEG [32]. The same group reported a case where the patient presented EDS and periodic leg movements that were related to epileptic discharges revealed only by stereo-EEG and that improved after surgical treatment [33]. In a prospective cohort of TLE patients EDS was evaluated before, 3 months, and 1 year after surgical treatment. Results showed decrease in ESS and PSQI scores postoperatively and analysis for nocturnal seizures was not statistically significant [34]. The effects of vagus nerve stimulation (VNS) were also studied and daytime vigilance after 6 months of VNS treatment (evaluated with MSLT and visual reaction time) with low intensity stimulus (≤1.5 mA) improved, with positive impact on QOL [35].

### 3.2. Evidence from Children-Based Studies

In children with epilepsy (CWE), like adults, questionnaire-based studies reveal that they are more likely to have sleep complaints than the general pediatric population [11, 36–39]. In Table 2, a summary of the studies addressing the relationship between epilepsy and EDS is displayed. A combination of nocturnal seizures disrupting sleep architecture, sedative medication, and the presence of primary sleep disorders may represent a part of the multifactorial nature of the problem [40, 41]. EDS and sleep initiating/maintaining complaints are the most reported issues [37, 39]. A study with 26 patients showed EDS, parasomnia, and sleep-disordered breathing (SDB) to be more frequent in the epilepsy group and also defined parasomnia and SDB as independent predictors of EDS, while epilepsy syndrome, AEDs, and seizure freedom were not statistically significant [36]. The role of EDS and behavioral impairment has also been studied in CWE and their parents. In a group of 97 CWE, the autistic spectrum disorder (ASD) was screened together with sleep disorders. The worst behavior and daytime sleepiness were seen in children with greater risk of having ASD, suggesting that behavioral difficulties and EDS could affect their ability to learn [42]. A recent study evaluated disrupted sleep in CWE and their parents (105 households and 79 controls) and reported high rates of parent-child room sharing and cosleeping in the epilepsy group. CWE had greater sleep disturbance (especially parasomnia, EDS, and sleep walking) and their parents had more fatigue and sleep dysfunction. Severity of epilepsy correlated with child and parent sleep dysfunction and also with parent fatigue [43]. Maternal depression has also been evaluated. In a study of 52 mothers of CWE, 45% had high scores on depression scales with 25% in the moderate/severe range. Maternal depression correlated with behavioral problems (attention deficit) but not with epilepsy [44].

The effect of medication (AED and other sleep-active drugs) on daytime sleepiness of CWE is also a matter of discussion. One of the most studied sleep-active drugs in children is melatonin. Melatonin can reduce excitability of glutamate secreting neurons and increase activity of GABAergic inhibitory neurons, and it also has metabolites that act as an anticonvulsant and a free radical scavenger. It has been suggested that melatonin may have an anticonvulsant effect [38, 45]. A study with 37 patients (23 with intractable seizures) addressed the role of melatonin in improving sleep-related complaints. Melatonin was associated with improvements in EDS and sleep-related complaints and also with significant reduction in seizure severity [38]. The role of AED in EDS of CWE is less straightforward. A study with 46 children taking valproic acid (VPA) assessed sleep duration and sleep behavior before and after tapering it in patients treated for more than 6 months. Subjective and objective (actigraphy) evaluation was performed at baseline, 8, and 12 week after treatment. Actigraphy data showed reduction in the average sleep without VPA in 33 children and increase in 13 children. Mean actual sleep time per day was reduced after VPA termination, but the reduction was only significant in children older than 6 years [46]. 

## 4. Conclusion

The relationship between EDS and epilepsy is still a matter of discussion. Although EDS in PWE is comparable to the general population, it carries more severe consequences in QOL and the presence of primary sleep disorders seems to contribute more to EDS symptoms than epilepsy-related factors. In CWE, EDS seems to contribute to parent-child cosleeping and to behavioral and learning disabilities. Only a few studies done in the last decade test the patients with subjective and objective evaluation, probably because of the logistic difficulties of performing PSG and MSLT in a bigger (population) set. Difficulties apart, EDS in epilepsy is a prosperous area of research and more knowledge is needed to understand (1) a better definition of EDS and fatigue in research methodology; (2) more studies combining subjective and objective evaluation; (3) the role of medication; (4) the role of psychiatric comorbidity; and (5) impact of treatment of coinciding primary sleep disorders.

## Figures and Tables

**Table 1:** Adult-based studies addressing EDS in PWE.

Author/place/year	Study design	Population	Instruments	Findings on EDS
Zanzmera et al. India, 2012 [17]	Prospective cohort	40 patients (20 with refractory and 20 with controlled epilepsy)	Medical history; sleep prestructured questionnaire; ESS; PSG.	ESS scores higher in The medically refractory group, but the number of patients with ESS > 10 was similar in both groups. Average TST is shorter in the refractory group in contrast to self-reported sleep. Also this group had more abnormal arousals, sleep efficiency, and awakenings. 20% in the refractory group with AHI > 5 and none in the controlled group.

Zhou et al. China, 2012 [27]	Prospective cohort	11 patients 10 controls	Athens Insomnia Scale; ESS; MSLT; PSG	Levetiracetam reduced TST and increased daytime sleepiness compared to baseline. Reduced REM sleep on PSG. Increase in ESS score but no changes in MSLT after levetiracetam treatment.

Krishnan et al. India, 2012 [16]	Cross-sectional	50 with JME, on VPA only, and 38 controls	Medical and family history and neurological examination. EEG, neuroimaging; ESS; PSQI; NIMHANS sleep disorders questionnaire.	ESS score ≥ 11 in 34% of patients. PSQI score ≥ 6 in 48% of patients. Mean ESS and PSQI scores significantly high in the JME group and a high prevalence of EDS and insomnia.

Chen et al. Taiwan, 2011 [15]	Cross-sectional	117 patients 30 controls	Medical record ESS; PSQI.	ESS score ≥ 10 in 20% of patients and 7% of controls. PSQI total scores were higher in patients (6.5 versus 3.7). Patients had higher sleep latency (1.2 versus 0.6).

Giorelli et al. Brazil, 2011 [19]	Cross-sectional	99 patients	Medical record; BNSQ; ESS; Beck Depression and Anxiety Inventory.	Prevalence of EDS (ESS > 10) was 47.5% and showed relationship with anxiety and neck circumference.

Venturi et al. Brazil, 2011 [20]	Cross-sectional	98 patients	Medical record; Berlin clinical questionnaire; ESS; Beck Depression and Anxiety Inventory.	Patients with epilepsy have a high risk of obstructive sleep apnea (55 %) which is related to neck circumference, anxiety, arterial hypertension, and high body mass index.

Carrion et al. Brazil, 2010 [34]	Prospective cohort	48 patients 43 controls	Seizure frequency scale; ESS; PSQI.	PSQI was high before epilepsy surgery and decreased after the procedure. ESS scores reduced after surgery, but no score was in the pathological range. TLE patients have poor subjective sleep quality that improves after epilepsy surgery.

Klobučníková et al. Slovak Republic, 2009 [18]	Cross-sectional	83 patients 80 controls	Medical record; ESS; PSG; MSLT.	ESS > 9 in 37.3% of patients and in 11.2% of controls. Mean ESS was higher in patients (7.1 versus 5.5). Patients had lower sleep efficiency and more stage N2 and less N3 and REM than controls. MSLT performed only in epilepsy group (mean latency 13 min.).

Kwan et al. China, 2009 [25]	Cross-sectional	247 patients	Hospital Anxiety and Depression Scale; Hamilton Anxiety Rating Scale; Medical Outcomes Study Sleep Scale; ESS; QOLIE-31.	Subjective anxiety, depression, and sleep disturbance correlated with QOLIE-31. High ESS score, polytherapy, psychiatric comorbidity and seizures in the last month were related with lower QOLIE-31 score.

Romigi et al. Italy, 2009 [30]	Prospective cohort	12 patients	ESS; blood chemistry (plasma level of pregabalin); EEG; PSG.	Pregabalin reduced seizures, increased REM sleep, and decreased stage 2 sleep. Significant increase in ESS score without reaching the pathological cut-off (ESS < 10).

Piperidou et al. Greece, 2008 [14]	Cross-sectional	124 patients	Medical record ESS; SA-SDQ; Athens Insomnia Scale; QOLIE-31.	Prevalence of EDS was 16.9%. 28.2% had OSA, based on SA-SDQ. No correlation with EDS and OSA. Prevalence of insomnia was 24.6%. Patients with EDS had lower score on QOLIE-31, and patients with OSA had lower score in cognitive functioning. Insomnia correlated with seizure frequency and was an independent predictor of reduced quality of life.

Wood et al. Canada, 2008 [44]	Cross-sectional	52 mothers of children with intractable epilepsy	Beck Depression Inventory; PSQI; Maternal Quality of Life; Impact of Pediatric Epilepsy Scale; Child Behavior Checklist; Attention Deficit Hyperactivity Disorder Rating Scale-IV; Scales of Independent Behavior-Revised.	45% of mothers scored as depressed (25% in the severe range). Sleep disruption in 67%. Maternal depression correlated with child behavior disorders, but not with epilepsy-related variables, autism, adaptive delay, or family income.

Chihorek et al. USA, 2007 [21]	Prospective cohort	21 (>50 years with late-onset or worsening seizures and seizure-free or/with improvement)	Medical record; ESS; SA-SDQ; PSG. CPAP treatment for patients diagnosed with OSA.	The late-onset group had higher AHI, ESS score, and SA-SDQ score, Mean ESS of 11.6 in the late-onset group and 5.9 in seizure-free group. Correlation between the ESS scores and AHI. Patients with CPAP treatment had improvements in seizure control and EDS.

Nobili et al. Italy, 2007 [32]	Case series	21 NFLE	ESS; video-EEG; stereo-EEG; MRI; PSG.	6 months surgery EDS was resolved in the 9 patients with this complaint preoperatively.

de Haas et al. The Netherlands, 2007 [31]	Double-blind randomized prospective cohort	17 patients (8 in placebo group and 9 in pregabalin group)	Medical Outcomes Study Sleep Scale; seizure diary; Groningen Sleep Questionnaire; Sleep Diagnosis List; PSG.	Pregabalin was associated with a reduction in the number of awakenings and improvement in wake time after sleep onset. It was also associated with improvements in sleep disturbance scales and sleep quantity subscales compared with placebo.

Vignatelli et al. Italy, 2006 [26]	Cross-sectional	33 NFLE 27 controls	ESS; Bologna questionnaire on sleepiness-related symptoms; questionnaire on nocturnal sleep quality; Berlin questionnaire.	EDS did not differ between groups. NFLE group being more “tired after awakening” (36% versus 11%) and having “spontaneous mid-sleep awakenings” (50% versus 22%).

Nobili et al. Italy, 2006 [33]	Case report	1 female patient with drug-resistant NFLE	ESS; video-EEG; stereo-EEG.	Periodic leg movements during sleep and EDS were associated with enhanced arousal instability induced by epileptic discharges not detectable on scalp EEG.

Höllinger et al. Switzerland, 2006 [22]	Cohort study	29 with OSA and epilepsy	ESS; PSG; CPAP.	In 21 patients, the appearance of OSA coincided with an increase in seizure frequency or status epilepticus. Four patients with good CPAP compliance had reduction of ESS scores and seizure frequency.

Khatami et al. Switzerland, 2006 [13]	Cross-sectional	100 patients 90 controls	Clinical interview; ESS; SA-SDQ; Ullanlinna Narcolepsy Scale.	Sleep complaints in 30% of patients and 10% of controls. Maintenance insomnia more frequent in patients (52% versus 38%). EDS, sleep apnea, and restless legs symptoms with no difference between groups.

Khatami et al. Switzerland, 2005 [28]	Case report	A 36-year old woman	ESS; MSLT; PSG; MRI; PET.	Present a severe but potentially reversible hypersomnia with levetiracetam as add-on treatment in epilepsy patients and consider objective sleep-wake tests in epilepsy patients complaining of daytime fatigue/somnolence.

de Weerd et al. The Netherlands, 2004 [12]	Cross-sectional	486 patients 492 controls	WHO epilepsy questionnaire; Sleep Diagnosis List; Medical Outcomes Study Sleep Scale; Groningen Sleep Questionnaire; ESS; SF-36 Health Survey.	Sleep disturbance is in patients more prevalent than controls (38.6% versus 18%). Patients had more EDS (relation observed with SDL but not with the ESS). Patients with more impairment in quality of life.

Bonanni et al. Italy, 2004 [29]	Prospective cohort	14 with focal epilepsy and 14 controls	ESS; MSLT; Simple and choice visual reaction times; ambulatory PSG.	No change in baseline ESS score and mean latencies in MSLT after 2 months of monotherapy with topiramate 200 mg/day.

de Almeida et al. Brazil, 2003 [24]	Cross-sectional	39 with temporal lobe epilepsy	Medical and sleep habits record; ESS; MSLT; PSG.	85% with EDS, 75% with seizures during sleep, and 26% with insomnia. Parasomnia was the most common sleep disorder (67%). PSG showed sleep fragmentation, reduction in REM sleep, and increase in wake time after sleep onset. ESS correlated with MSLT.

Weatherwax et al. USA, 2003 [23]	Cross-sectional	125 patients	SA-SDQ; PSG.	45% had AHI > 5. SA-SDQ score of 29 with 75% sensitivity and 65% specificity for men. Score of 26 with 80% sensitivity and 67% specificity for women.

Galli et al. Italy, 2003 [35]	Prospective cohort	8 patients	Visual analog scale of quality of life; visual reaction times; MSLT.	VNS at low intensities stimulus improved daytime vigilance in epileptic patients, with an improvement of quality of life.

Manni et al. Italy, 2000 [3]	Cross-sectional	244 cases, 205 controls	Medical record; ESS; Sleep Habits Questionnaire.	ESS score was similar in both groups (5.6 versus 5.7). ESS score > 10 in 11% of patients and 10% of controls.

Malow et al. USA, 1997 [2]	Cross-sectional	158 cases, 68 controls	ESS; SA-SDQ; PSG in only 27 patients.	28% of patients and 18% of controls with ESS score > 10. SA-SDQ scores and RLS were independent predictors of ESS score > 10.

ESS: Epworth sleepiness scale; TST: total sleep time; AHI: apnea-hypopnea index; EEG: electroencephalography; PSG: polysomnography; PET: positron emission tomography; PSQI: Pittsburgh sleep quality index; JME: juvenile myoclonic epilepsy; VPA: valproic acid; BNSQ: basic nordic sleep questionnaire; MSLT: multiple sleep latencies test; REM: rapid eye movement; SA-SDQ: sleep apnea section of the sleep disorders questionnaire; QOLIE-31: quality of life in epilepsy; OSA: obstructive sleep apnea; CPAP: continuous positive airway pressure; NFLE: nocturnal frontal lobe epilepsy; WHO: World Health Organization.

**Table 2:** Children-based studies on epilepsy and EDS.

Author/place/year	Study design	Population	Instruments	Findings on EDS
Larson et al. USA, 2012 [43]	Cross-sectional	105 households with a child with epilepsy 79 controls	E-Chess; CSHQ; PSQI; Iowa Fatigue Scale.	Increased rates of room sharing and cosleeping in epilepsy group. Parents of children with epilepsy with more sleep dysfunction and fatigue. Children with epilepsy with more daytime sleepiness, parasomnias, bedtime resistance, and sleep onset delay.

Tang et al. UK, 2011 [39]	Cross-sectional	43 patients 494 controls	CSHQ	CHSQ was higher in the epilepsy group and parents reported shorter sleep duration, parasomnias, and increased daytime sleepiness in the rolandic epilepsy group.

Elkhayat et al. Egypt, 2010 [38]	Prospective clinical trial	37 patients 23 with intractable epilepsy (received oral melatonin as the study intervention)	Medical record; CSHQ; ESS-pediatric version; EEG; laboratorial analysis (serum melatonin level).	Intractable seizures group with higher scores in sleep walking, bruxism, and OSA than controlled seizures. No difference in EDS between groups. ESS score and EDS improved after melatonin administration. 87% of the melatonin group improved seizure frequency and severity. 13% had worsening of seizures and melatonin had to be withdrawn.

Ong et al. Malaysia, 2010 [37]	Cross-sectional	92 cases; 92 healthy siblings as controls	Medical record; SDSC; EISS; ESSS-C.	Higher SDSC score in the epilepsy group in disorders of initiating and maintaining sleep, sleep-wake transition, sleep-disordered breathing, and EDS. Epilepsy group with higher prevalence of cosleeping. Higher EISS scores were associated with higher total SDSC scores.

Schmitt et al. Switzerland, 2009 [46]	Prospective single-blinded clinical trial	46 patients	Medical record; modified version of the Sleep Habits Survey; sleep diary; actigraphy.	Questionnaire data showed no difference before and after ending VPA treatment. 33 had average sleep time reduced and it increased in 13 children. VPA withdrawal showed significant reduction in sleep duration in children older than 6 years.

Batista and Nunes Brazil, 2007 [11]	Cross-sectional	121 cases, 121 controls	Sleep Habits Inventory for Preschool Children; Sleep Behavior Questionnaire.	Worse sleep habits in children with epilepsy were associated with nocturnal seizures, polytherapy, developmental delay, refractory seizures, generalized seizures, and epileptic syndromes with an unfavorable outcome.

Maganti et al. USA, 2006 [36]	Cross-sectional	26 cases, 26 controls	Pediatric Sleep Questionnaire; Pediatric Daytime Sleepiness Scale.	EDS, parasomnias, and sleep-disordered breathing were more reported in the epilepsy group. In PDSS, EDS scores were worse in the epilepsy group. Parasomnias and sleep-disordered breathing were independent predictors of EDS in the epilepsy group.

Clarke et al. USA, 2005 [42]	Cross-sectional	97 patients	Medical record; Autism Screening Questionnaire; Pediatric sleep Questionnaire;	32% of children with epilepsy fit the criteria for ASD. Worst behavior and EDS were present in those at greater risk. Seizures occurred earlier in children at risk of ASD. Behavioral difficulties and EDS could affect the ability to learn.

Becker et al. USA, 2004 [41]	Cross-sectional	30 patients	Medical record; PPVT-III, CPRS-R:L; ECBI; Children's Depression Inventory; Revised Children's Manifest Anxiety Scale; Pediatric Sleep Questionnaire; PSG.	80% had sleep disruption because of either OSA, disturbance of sleep architecture, or sleep fragmentation. Daytime behavior problems may be attributed to specific disruptions in sleep regulations.

Becker et al. USA, 2003 [40]	Cross-sectional	14 cases, 14 controls	Pediatric Sleep Questionnaire; CPRS-R:L; ECBI; Children's Depression Inventory; Revised Child Manifest Anxiety Scale; PSG.	More than 50% of children with epilepsy had behavioral problems. No difference in snoring, EDS, and restless sleep. Sleep disturbance may be related to behavioral problems in children with epilepsy.

CSHQ: children's sleep habits questionnaire; PSQI: Pittsburgh sleep quality index; E-Chess: early childhood epilepsy severity scale; PSG: polysomnography; EDS: excessive daytime sleepiness; SDSC: sleep disturbance scale for children; ASD: autistic spectrum disorder; PPVT-III: peabody picture vocabulary test-third edition; CPRS-R:L: Connor's parent rating scale-Revised long form; ECBI: Eyberg child behavior inventory; EISS: epilepsy illness severity score; ESSS-C: Epilepsy syndrome severity score-child; ESS: Epworth Sleepiness scale; VPA: valproic acid; AED: antiepileptic drug.
